# Immune escape mechanisms and immunotherapy of urothelial bladder cancer

**Published:** 2021-07-30

**Authors:** Zhao Yang, Yinyan Xu, Ying Bi, Nan Zhang, Haifeng Wang, Tianying Xing, Suhang Bai, Zongyi Shen, Faiza Naz, Zichen Zhang, Liqi Yin, Mengran Shi, Luyao Wang, Lei Wang, Shihui Wang, Lida Xu, Xin Su, Song Wu, Changyuan Yu

**Affiliations:** ^1^Department of Biomedical Engineering, College of Life Science and Technology, Beijing University of Chemical Technology, Beijing 100029, China; ^2^Department of Bioscience, College of Life Science, Key Laboratory of Protection and Utilization of Biological Resources in Tarim Basin of Xinjiang Production and Construction Corps, Tarim University, Alar 843300, Xinjiang, China; ^3^Department of Urology, The Affiliated Luohu Hospital of Shenzhen University, Shenzhen University, Shenzhen 518000, China; ^4^Department of Urology, The Second Affiliated Hospital of Kunming Medical University, Kunming 650101, China; ^5^Department of Urology, Xuanwu Hospital, Capital Medical University, Beijing 100053, China

**Keywords:** urothelial bladder cancer, immune escape, immunotherapy, immune checkpoints, immune cells

## Abstract

**Background and aim:**

Urothelial bladder cancer (UBC) is a common malignant tumor of the urogenital system with a high rate of recurrence. Due to the sophisticated and largely unexplored mechanisms of tumorigenesis of UBC, the classical therapeutic approaches including transurethral resection and radical cystectomy combined with chemotherapy have remained unchanged for decades. However, with increasingly in-depth understanding of the microenvironment and the composition of tumor-infiltrating lymphocytes of UBC, novel immunotherapeutic strategies have been developed. Bacillus Calmette-Guerin (BCG) therapy, immune checkpoint blockades, adoptive T cell immunotherapy, dendritic cell (DC) vaccines, etc., have all been intensively investigated as immunotherapies for UBC. This review will discuss the recent progress in immune escape mechanisms and immunotherapy of UBC.

**Methods:**

Based on a comprehensive search of the PubMed and ClinicalTrials.gov database, this review included the literature reporting the immune escape mechanisms of UBC and clinical trials assessing the effect of immunotherapeutic strategies on tumor or immune cells in UBC patients published in English between 1999 and 2020.

**Results:**

Immune surveillance, immune balance, and immune escape are the three major processes that occur during UBC tumorigenesis. First, the role of immunosuppressive cells, immunosuppressive molecules, immunosuppressive signaling molecules, and DCs in tumor microenvironment is introduced elaborately in the immune escape mechanisms of UBC section. In addition, recent progress of immunotherapies including BCG, checkpoint inhibitors, cytokines, adoptive T cell immunotherapy, DCs, and macrophages on UBC patients are summarized in detail. Finally, the need to explore the mechanisms, molecular characteristics and immune landscape during UBC tumorigenesis and development of novel and robust immunotherapies for UBC are also proposed and discussed.

**Conclusion:**

At present, BCG and immune checkpoint blockades have been approved by the US Food and Drug Administration for the treatment of UBC patients and have achieved encouraging therapeutic results, expanding the traditional chemotherapy and surgery-based treatment for UBC.

**Relevance for patients:**

Immunotherapy has achieved desirable results in the treatment of UBC, which not only improve the overall survival but also reduce the recurrence rate and the occurrence of treatment-related adverse events of UBC patients. In addition, the indicators to predict the effectiveness and novel therapy strategies, such as combination regimen of checkpoint inhibitor with checkpoint inhibitor or chemotherapy, should be further studied.

## 1. Introduction

Ranked the tenth most common malignant tumor worldwide, urothelial bladder cancer (UBC) is a major threat to public health, with approximately 573,728 new cases and about 212,536 deaths in 2020 [1]. UBC has a high incidence in European countries and North America, and is more prevalent in males than in females [2,3]. The initiation and development of UBC are sophisticated and multifactorial processes. The main pathogenic factors in UBC are cigarette smoking, exposures to aromatic carcinogens, bladder infection and inflammation [2]. An epidemiological study found that UBC-related mortality rates were higher among urban residents than those among rural residents, resulting from the long exposure to tobacco and the chemical industry [3].

UBC was first recognized by Lacuna in 1551 [4]. According to the histologic origin, UBC are divided into transitional epithelial carcinoma (TCC), squamous cell carcinoma, adenocarcinoma, small cell carcinoma, etc. TCC is the predominant form of UBC, which can be clinically categorized into non-muscle-invasive bladder cancer (NMIBC) and MIBC. About 70% of TCC patients are diagnosed with NMIBC, which has a good prognosis, although about 31–78% of NMIBC patients relapse within 5 years [5]. By contrast, about 30% of UBC patients are diagnosed with MIBC, which has a poor prognosis and a mortality rate of approximately 50% [5,6]. At present, transurethral resection of bladder tumor (TURBT), radical cystectomy, neoadjuvant systematic chemotherapy, and intravesical chemotherapy are the main treatment options for UBC [7,8]. However, above standard therapies have remained unchanged for three decades [3]. As the high rate of recurrence and the need for long-term surveillance greatly increased the economic burden of UBC patients [9], there is an urgent need to explore novel therapies.

**Figure 1:**
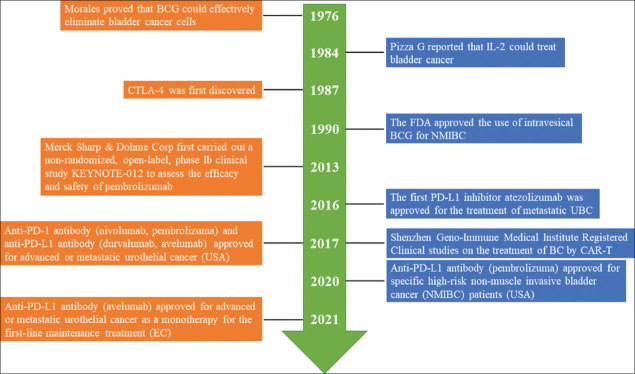
Development of immunotherapies for urothelial bladder cancer

Immunotherapy has revolutionized cancer treatment in recent years, which enhances or inhibits the immune function of the body to achieve the purpose of the treatment of diseases. The development of immunotherapy for UBC is shown in Figure 1. In 1976, Morales *et al*. first reported the treatment of UBC with Bacillus Calmette-Guerin (BCG) [10]. Furthermore, Lamm *et al*. confirmed the effect of BCG in the treatment of UBC in 1980 [11]. In addition, more evidence proved that BCG is an effective biological immunotherapy in treating carcinoma *in situ*, preventing tumor progression and post-operative recurrence, and improving survival rate of UBC patients [12,13]. Moreover, the first programmed cell death 1 (PD-1) ligands (PD-L1) inhibitor atezolizumab was approved for the treatment of metastatic UBC in 2016 [14]. Then, in 2017, the US Food and Drug Administration (FDA) approved additionally four immune checkpoint drugs for the treatment of UBC. Specifically, nivolumab and avelumab were approved for the treatment of locally advanced or metastatic UBC on February 2 [15] and May 9 [16], respectively. Furthermore, durvalumab developed by AstraZeneca received accelerated approval from the FDA for the treatment of patients with locally advanced or metastatic UBC after failure of a platinum-containing regimen on May 1 [17]. Based on the results of the KEYNOTE-045 test, the FDA also approved pembrolizumab for certain locally advanced or metastatic UBC patients on May 18 [18]. Furthermore, pembrolizumab was also approved by the FDA on January 8, 2020 for the treatment of NMIBC patients, which is the first PD-1 inhibitor approved for the treatment of specific high-risk NMIBC patients [19]. Finally, the European Commission (EC) has approved the anti-PD-L1 therapy avelumab as a monotherapy for the first-line maintenance treatment of adult patients with locally advanced or metastatic UBC who have not progressed after receiving first-line platinum-containing chemotherapy in 2021 [20].

UBC represents an ideal disease state to study immune evasion and mechanisms by which to improve the immune response based on several established features. With in-depth understanding of the microenvironment of UBC and the composition of tumor-infiltrating lymphocytes, immunotherapy has led to breakthroughs in the treatment of UBC. The treatments includes BCG therapy, immune checkpoints inhibitors, tumor vaccines, and adoptive immunotherapy. [21,22]. Furthermore, the Cancer Genome Atlas (TCGA) study found that genes regulating chromatin remodeling were more frequently mutated in UBC than in other type of cancer, which may represent the additional targets for novel therapies in combination with immunotherapy. This review will describe the immune escape mechanisms of UBC and highlight the research progress and clinical development of UBC immunotherapies.

## 2. Immune Escape Mechanisms of UBC

During the occurrence and development of UBC, immune surveillance, immune balance, and immune escape are the three major processes. The research on the mechanisms of immune escape in UBC is helpful to design new approaches for the immunotherapy. Accumulating evidence shows that UBC cells evade immunosurveillance by orchestrating complex immunosuppressive networks in the tumor microenvironment. The major immunosuppressive regulations are as follows:

### 2.1. Recruitment of immunosuppressive cells in tumor microenvironment

There are many kinds of immunosuppressive cells in tumor microenvironment, and myeloid-derived suppressor cells (MDSCs), regulatory T cells (Tregs), and M2-type tumor associated macrophages (TAMs) were reported to be functional in the microenvironment of UBC.

#### 2.1.1. Myeloid-derived suppressor cells

MDSCs are a heterogeneous population of immature myeloid cells that are recruited to the primary tumor as well as metastatic sites and play a crucial role in inhibiting innate and adaptive immune responses by suppressing CD4^+^ T cells, CD8^+^ T cells, and natural killer (NK) cells [23]. The peripheral blood and the tumor tissues isolated from UBC patients showed an increased amount of MDSCs compared to healthy donors or normal bladder tissues, respectively [24,25]. Specifically, MDSCs identified in the peripheral blood of UBC patients were composed by granulocytic CD15high CD33low HLA-DRneg and monocytic CD15low CD33high HLA-DRneg MDSCs, which produced substantial amounts of CCL2, CCL3, CCL4, G-CSF, interleukin-8 (IL-8), and IL-6. Moreover, the MDSCs isolated from the peripheral blood of UBC patients could activate CD4^+^Foxp3^+^ Tregs cells and inhibit the T cell proliferative response.

Within the tumor microenvironment, the secretion of pro-inflammatory cytokines such as vascular endothelial growth factor (VEGF), IL-1, and IL-6 directs the differentiation of immature myeloid cells into pro-tumorigenic MDSCs through inhibition of cytotoxic T-cells (CTLs) function [26]. Immunohistochemical analysis of UBC tissues revealed elevated levels of CD33^+^ MDSCs, due to increased recruitment of MDSCs in the tumor microenvironment mediated by the CXCL2/MIF-CXCR2 axis. This tumor-promoting axis demonstrates strong suppression of T-cell proliferation and is significantly associated with advanced disease stage and poor prognosis [27].

The results of *in vivo* experiments indicated that the human UBC cells SW780 and Urothel 11, and murine UBC cells MBT2 could recruit the host’s myeloid cells, including MDSCs and macrophages [14,28]. In the tumors formed by SW780 or Urothel 11 in immunodeficient mice model, the tumor-infiltrating MDSCs were mostly composed by MHC class II-positive F4/80^+^ macrophages and Ly6C^+^F4/80^+^ macrophage precursors, or Ly6c^+^F4/80^−^ MDSCs and Ly6C^+^F4/80^+^ macrophage precursors, respectively. In addition, Gr-1^+^ MDSCs could differentiate into highly immunosuppressive PD-L1^+^ macrophages induced by PGE2 producing MBT2 cells [27].

Taken together, UBC correlated with an enhanced number of MDSCs in both the peripheral blood and tumor tissue. The size, growth rate, and subtypes of the tumors may correlate the composition and numbers of MDSCs infiltrated in the peripheral blood and the tumor tissues isolated from UBC patients. Mechanically, the pro-inflammatory and immunosuppressive cytokines/chemokines secreted by the MDSCs promotes cancer-related inflammation and immune evasion.

#### 2.1.2. Tregs

The role of Tregs is to dampen chronic immune responses against viruses, tumors and self-antigens. Frequently defined by the expression of CD4^+^ and FOXP3^+^, Tregs have a high frequency in tumor and are correlated with poor outcomes in cancer patients. In UBC, the number of Tregs was significantly elevated in both peripheral blood and tumor tissues, which inversely correlated with recurrence-free survival [29]. Horn *et al*. showed that Tregs in UBC competed with CD4^+^ T effector cells through expression of identical tumor-associated antigens (TAAs). Thus, TAA-specific T cell responses against these antigens are suppressed by Tregs [30]. In a recently published study, the level of Tregs in human bladder tissue significantly correlated with both TAMs and IL-6-positive cancer cell count [31]. Therefore, suppressing these populations of immunosuppressive cells could improve the treatment of UBC patients.

#### 2.1.3. Tumor-associated macrophages

TAMs are inflammatory cells found in malignant tumors that play important roles in tumor growth, progression, and metastases. TAMs may induce angiogenesis by secreting pro-angiogenic molecules [32], eliminate CD8^+^ T cells [33,34], support the induction and transportation of Tregs [35-37] through secreting immunosuppressive cytokines and bioactive lipids [38,39]. Furthermore, augmented infiltration of TAMs in UBC patients correlated with the resistance to BCG immunotherapy [40] and poor prognosis after intravesical instillation of BCG [31]. Altogether, TAMs may directly interfere with the BCG induced immune response and may represent a surrogate marker for BCG resistance.

### 2.2. Upregulation of immunosuppressive molecules

Tumor cells evade immunosurveillance through the elevation of co-inhibitory/stimulatory PD-L1 or B7 ligands, which binds to inhibitory PD-1 or CTLA-4 receptors on T cells and resulted in the inhibition of anti-tumor immunity and exhaustion of T cells [41]. Qian *et al*. found that toll-like receptor 4 (TLR4) signaling pathway induced the expression of PD-L1 in UBC cells and facilitated the evasion of immune surveillance by UBC cells, which process could be suppressed by ERK or C-Jun N-terminal kinases inhibitors [42]. Furthermore, gene expression studies demonstrated that the expression of Fas ligand (FasL) in UBC patients was higher than those of healthy individuals, regardless of grading, and staging of tumors [43]. FasL-expressing UBC cells can induce Fas-mediated killing of autologous T lymphocytes both *in vitro* and *in vivo*. These findings suggested that FasL acted as an important regulator of immune escape through the induction of T cell apoptosis [43].

### 2.3. Secretion of immunosuppressive signaling molecules

Inducible inflammatory enzyme COX2 was highly expressed in UBC cells and promoted the secretion of PGE2, the major metabolite of the COX pathway [44,45]. PGE2 plays multifaceted roles in cancer progression, cancer-related immune inflammation, and immune evasion, which exhibits strong anti-apoptotic effects, induces resistance to chemotherapy, supports proliferation, and renewal of UBC stem cells [46]. High levels of PGE2 in tumor tissue has a strong impact on the function of infiltrating immune cells (ICs) including the inhibition of antigen presenting cells (APCs) and effector T cells, and the stimulation of MDSCs generation directly through PGE2-specifc EP2 and EP4 receptors. UBC and tumor-infiltrating inflammatory cells in advanced tumors were positive for COX2 and exhibited increased expression of another PGE2-producing enzyme, mPGES1 [47]. Moreover, Eruslanov *et al*. [24] demonstrated that human bladder tumors secreting PGE2 markedly inhibited the generation of mature APCs *in vitro*, while promoting the accumulation of monocytic MDSCs and macrophages. Altogether, these data indicated that deregulated PGE2 metabolism in the UBC promoted the formation of immunosuppressive tumor-supporting microenvironment.

Tumor-promoting signaling molecules including transforming growth factor-β (TGF-β), IL-10, IDO, and VEGF inhibited tumor antigen presentation, as well as activation, proliferation, differentiation, and cytotoxicity of T cells, thereby limiting the efficacy of anti-tumor immunity [48]. Yang and Lattime found that IL-10-expressing UBC cell line MB4 suppressed the ability of dendritic cells (DCs) to stimulate CD4^+^ and CD8^+^ T cell anti-tumor responses [49]. Furthermore, Loskog *et al*. demonstrated that CD40L-transduced MB49 cells suppressed the production of IL-10 and TGF-β, which promoted the maturation and activation of DCs, and induced a Th1-type response and the activation of CTLs in the tumor area. These results suggested that immunosuppressive signaling molecules were the possible candidates for the treatment of UBC.

### 2.4. DCs

DCs also contributed significantly to the tumorigenesis of UBC. Troy *et al*. reported that tumor-infiltrating DCs in UBC tissue were mainly immature and significantly fewer in number compared with those in normal bladder tissue. The low infiltration and functional deficiency of DCs resulted in non-effective antigen presentation, and the expression of costimulatory and adhesion molecules were too low to induce a specific CTLs response, which eventually led to immune escape of UBC [50].

## 3. Immunotherapy of UBC

### 3.1. BCG

BCG is a live, slow-growing, attenuated form of *Mycobacterium bovis*, which was discovered by French scientists Albert Léon Charles Calmette and Camille Guérin. Previously, it was used as a vaccine for newborns to prevent tuberculosis [51]. At present, BCG is the gold-standard intravesical immunotherapy for the treatment of NMIBC. The potential of BCG to treat UBC was discovered by Morales *et al.*, who successfully injected BCG into the bladder for the treatment of recurrent superficial UBC in 1976 [10] (Figure 1).

Encouraging data suggest that BCG treatment reduces long-term tumor relapse rate, tumor progression, tumor metastasis, and mortality of UBC patients. For example, a meta-analysis showed that intravesical instillation of BCG combined with TURBT could reduce the risk of UBC recurrence compared with TURBT alone [52]. In patients with medium- or high-risk Stage Ta and T1 UBC, combined TURBT and BCG treatment led to a reduction of approximately 56% in recurrence rate compared with TURBT alone [52]. Furthermore, clinical studies have shown that the recurrence rate with BCG perfusion and continuous treatment was about 32% lower than that achieved with anti-tumor antibiotic mitomycin C [53]. Finally, BCG also reduced the risk of progression from NMIBC to MIBC [54]. Regarding safety, BCG is well tolerated and no grade 3 or 4 toxicity has been reported [55]. Based on these findings, the FDA approved intravesical BCG instillation for treatment of NMIBC in 1990 [44,56] (Figure 1). At present, the European Association of Urology, American Urological Association, and Urological Guidelines of China provide comprehensive guidance for BCG as a standard treatment for intermediate- and high-risk NMIBC patients [57].

Mechanically, BCG could generate oxidative stress in UBC cells, lead to cell apoptosis and necrosis of UBC cells, and induce the immune response in the host [58]. First, BCG may activate the TLR7 and the following caspase 8 signaling pathway in UBC cells, which initiated the extrinsic apoptosis pathway of UBC cells [59]. Another study demonstrated that BCG could also increase the expression of lysosomal hydrolase cathepsin B and activate pro-apoptotic protein BID and pro-caspase 9 in UBC cells, which initiated the intrinsic apoptosis pathway of UBC cells [60]. Besides apoptosis, BCG led to the caspase-independent cell membrane integrity damage, ultrastructural changes, and the release of necrosis associated chemokine high molecular group box protein 1 [61]. Second, BCG induced the generation of nitric oxide synthase (iNOS) [62,63] or reactive oxygen species such as hydrogen peroxide (H_2_O_2_) [64], which both produced NO. This process led to the damage of DNA and proteins in UBC cells, causing cell apoptosis and autophagy ultimately [65]. Finally, BCG could activate nuclear factor kappa-B and promote the transcription of cytokines, which participated in the immune response [66,67]. BCG and the released cytokines could also activate CD8^+^ CTLs, macrophages, neutrophils, NK cells, and others effector cells to kill tumor cells in distinct manners [68,69].

### 3.2. Checkpoint inhibitors

T cells are derived from hematopoietic stem cells of the bone marrow [39]. These naïve T cells migrate to the thymus for further differentiation and activation. As tumor cells could evade host immunity through expression of immune checkpoint molecules, notably PD-L1 and B7-1/2, immune checkpoint blockades using monoclonal antibodies is a potential therapeutic strategy to prevent immune escape by UBC cells, thereby reactivating T cells and impeding tumor growth [70]. Here, we discuss some interventions that harness T cells to improve anti-tumor response [31].

#### 3.2.1. PD-1/PD-L1

In normal tissue, the PD-1/PD-L1 axis attenuates T cell response and minimizes tissue damage on activation of pro-inflammatory cytokines. In cancer, PD-L1 expressing tumor cells could bind to the PD-1 receptors expressed in T cells. This neutralizes the anti-tumor effects of T cells through induction of apoptosis and exhaustion of CTLs, leading to immune escape of tumor cells [40,41,71-75]. Five immunotherapy agents targeting the PD-1 or PD-L1 pathway, namely pembrolizumab, nivolumab, atezolizumab, durvalumab, and avelumab, have been approved by the FDA for patients who have progressed during or after platinum-based therapy and have not received prior immunotherapy (Table 1).

**Table 1:** Summary results of studies of PD-1/PD-L1 immune checkpoint inhibitors in patients with urothelial bladder cancer

Immune checkpoint inhibitor	Target	Trial name	Study type	ORR	Median OS	Progression-free survival	Grade 3/4 adverse events	References
Pembrolizumab	PD-1	KEYNOTE-012 (NCT01848834) KEYNOTE-045 (NCT02256436)	Phase 1b Phase 3	25.9% Follow-up of 14.1 months: Chemotherapy group: 11.4% Pembrolizumab group: 21.1% Follow-up of 27.7 months: Chemotherapy group: Median 2-year: 14.3% Pembrolizumab group: Median 2-year: 26.9%	12.7 months Follow-up of 14.1 months: Chemotherapy group: 7.4 months Pembrolizumab group: 10.3 months Follow-up of 27.7 months: Chemotherapy group: 7.3 months Pembrolizumab group: 10.1months	2.0 months Follow-up of 14.1 months: Chemotherapy group: 3.3 months Pembrolizumab group: 2.1 months Follow-up of 27.7 months: Chemotherapy group: 3.3 months Pembrolizumab group: 2.1 months	15% Follow-up of 14.1 months: Chemotherapy group: 49.4% Pembrolizumab group: 15.0% Follow-up of 27.7 months: Chemotherapy group: 50.2% Pembrolizumab group: 16.5%	[76] [77,78]
		KEYNOTE-052 (NCT02335424)	Phase 2	29%	11.3 months	/	5%	[79]
		KEYNOTE-361 (NCT02853305)	Phase 3	Chemotherapy group: 44.9% Pembrolizumab group: 30.3% Combination group: 54.7%	Chemotherapy group: 14.3 months Pembrolizumab group: 15.6 months Combination group: 17.0 months	Chemotherapy group: 7.1 months Pembrolizumab group: 3.9 months Combination group: 8.3 months	/	[81]
Nivolumab	PD-1	CheckMate 032 (NCT01928394)	Phase 1/2	24.4%	9.7 months	2.8 months	21.8%	[99]
		CheckMate 275 (NCT02387996)	Phase 2	Follow-up of 6 months: All patients: 19.6% PD-L1 > 5%: 28.4% PD-L1 > 1%: 23.8% PD-L1 < 1%: 16.1% Follow-up of 33.7 months: All patients: 20.7%	Follow-up of 6 months: All patients: 8.74 months PD-L1 > 1%: 11.3 months PD-L1<1%: 5.95 months Follow-up of 33.7 months: 8.6 months	Follow-up of 6 months: 2 months Follow-up of 33.7 months: 1.9 months	18%	[28,84]
Atezolizumab	PD-L1	NCT01375842	Phase 1	Follow-up of 6 weeks: IHC 2/3: 43%; IHC 0 or 1: 11% Follow-up of 12 weeks: IHC 2/3: 52%	/	/	4%	[71]
		IMvigor210 (NCT02108652)	Phase 2	Cohort 1: 23% Cohort 2: All patients: 15% IC2/3: 27% IC1/2/3: 18%	Cohort 1: 15.9 months Cohort 2: All patients: 7.9 months IC2/3: 11.4 months IC1/2/3: 8.8 months	Cohort 1: 2.7 Cohort 2: 2.1 months	Cohort 1: 16% Cohort 2: 16%months	[86,87]
		IMvigor211 (NCT02302807)	Phase 3	In the IC2/3 population: Chemotherapy group: 22% Atezolizumab group: 26%	In the IC2/3 population: Chemotherapy group: 10.6 months Atezolizumab group: 11.1 months	In the IC2/3 population: Chemotherapy group: 4.2 months Atezolizumab group: 2.4 months In the intention-to-treat population: Chemotherapy group: 4.0 months Atezolizumab group: 2.1 months	In the intention-to-treat population: Chemotherapy group: 43% Atezolizumab group: 20%	[88]
Durvalumab	PD-L1	NCT01693562	Phase 1/2	Follow-up of 4.3 months: 31% Follow-up of 5.78 months: 17.8%	Follow-up of 5.78 months: 18.2 months	Follow-up of 5.78 months: 1.5 months	Follow-up of 4.3 months: 4.9% Follow-up of 5.78 months: 6.8%	[89,90]
Avelumab	PD-L1	NCT01772004 NCT02603432	Phase 1b Phase 3	18.2% 16.5%	13.7 months Control group: 14.3 months Avelumab group: 21.4 months	11.6 weeks Control group: 2.0 months Avelumab group: 3.7 months	6.8% Control group: 25.2% Avelumab group: 47.4%	[91] [92]

3.2.1.1. Pembrolizumab

Pembrolizumab is a monoclonal antibody targeting the PD-1 receptor initially approved for advanced melanoma, which could prolong OS with less toxicity and improved quality of life compared to additional lines of chemotherapy. In 2013, Merck Sharp & Dohme Corp. first carried out a non-randomized, open-label, phase Ib clinical study KEYNOTE-012 (NCT01848834, Figure 1) to assess the efficacy and safety of pembrolizumab [76]. After a median follow-up of 13 months, seven of 27 assessable patients showed significant overall response (OR). The median progression-free survival (PFS) and OS were 2.0 and 12.7 months, respectively. However, 53% of UBC patients experienced drug-related adverse reactions, and three patients experienced five serious treatment-related adverse events (TRAEs).

In another randomized phase III KEYNOTE-045 trial (NCT02256436) [77,78], 542 participants who had locally advanced. Metastatic or unresectable UBC that recurred or progressed after a platinum-based therapy were randomized to receive pembrolizumab 200 mg intravenous (IV) every 3 weeks or chemotherapy (paclitaxel, docetaxel, or vinflunine). The median 1- and 2-year OS rates of pembrolizumab group were 44.2% and 26.9%, respectively, which were higher than those in chemotherapy group (29.8% and 14.3%, respectively). In addition, the ORR was also higher in pembrolizumab group (21.1%) compared to chemotherapy group (11.0%). Moreover, pembrolizumab prolonged the OS of patients with advanced UBC to 10.3 months, compared with 7.4 months in those who received chemotherapy. As a second-line therapy for platinum-refractory advanced UBC, pembrolizumab also exhibited a lower rate of TRAEs than chemotherapy [77].

The effect of pembrolizumab was also examined in the first-line therapy. For example, the phase II KEYNOTE-052 (NCT02335424) study recruited 370 advanced UBC patients who were not suitable for cisplatin-based therapy and treated with 200 mg pembrolizumab every 3 weeks for up 2 years [79]. The ORR was 29% for the entire cohort, including 9% complete response and 20% partial response. The median duration of response was 30 months, with a median OS of 11.3 months [79,80]. In another large phase III trial, KEYNOTE-361 trial (NCT02853305) [81], the effect of pembrolizumab as a monotherapy was compared with chemotherapy with gemcitabine and cisplatin or carboplatin versus chemotherapy with pembrolizumab followed by maintenance pembrolizumab [82]. Approximately 1010 patients with advanced UBC were recruited and randomized in 1:1:1 fashion. The ORR of the combination group was 54.7%, which was better than those of chemotherapy group (44.9%) or pembrolizumab group (30.3%). Moreover, the median PFS of the combination group was 8.3 months, which was better than those of chemotherapy group (7.1 months) or pembrolizumab group (3.9 months). In addition, the median OS of the combination group was 17.0 months, which was also better than those of chemotherapy group (14.3 months) or pembrolizumab group (15.6 months).

Although the results of KEYNOTE-361 have dampened the enthusiasm regarding the pembrolizumab as a first-line therapy solely, the combination with chemotherapy of other agents provided a promising direction. Recently, the effect of another agent with antibody drug conjugate enfortumab and pembrolizumab was examined in EV-103 study including first-line cisplatin-ineligible cohort of 45 patients [83]. The ORR was 73.3% (95% confidence interval [CI], 58.1, 85.4) and seven patients with liver metastasis had a response rate of 53.3%, showing this potent combination.

3.2.1.2. Nivolumab

Nivolumab is another monoclonal antibody directed against PD-1. In a Phase II, single-arm, open-label CheckMate 275 study (NCT02387996) [84], 270 patients with metastatic or unresectable locally advanced UBC were enrolled to receive nivolumab 3 mg/kg IV every 2 weeks until measured disease progression, clinical deterioration, or unacceptable toxicity. Tumor PD-L1 expression was also quantified as ≥5% or ≥1%. With the minimum follow-up of 33.7 months, the ORR, median PFS, and median OS (95% CI) were 20.7% (16.1-26.1), 1.9 months (1.9–2.3), and 8.6 months (6.1–11.3) in all patients, respectively. In addition, the higher tumor mutational burden was associated (*P*<0.05) with improved ORR (OR [95% CI]: 2.13 [1.26–3.60]), PFS (HR: 0.75 [0.61–0.92]), and OS (HR: 0.73 [0.58–0.91]). These results indicated that nivolumab was clinical benefit with satisfactory safety profile and was initially approved by the FDA in February of 2017 [15].

3.2.1.3. Atezolizumab

Atezolizumab is a IgG1 monoclonal antibody and inhibits the interactions between the PD-1 and PD-L1 receptors through targeting PD-L1 protein [85]. In a Phase I study (NCT01375842), Powles *et al*. showed that atezolizumab (MPDL3280A) had noteworthy activity against metastatic UBC. Responses were rapid and occurred at the time of the first response assessment (42 days) [71], with an ORR of 35%. MPDL3280A was well tolerated, with a 4% rate of Grade 3 immune-related adverse effects [71].

In a Phase II, single-arm IMvigor210 clinical trial (NCT02108652) [14], the effect of atezolizumab toward UBC was examined in two separate cohorts. Participants in both cohorts will be given a 1200 mg IV dose of atezolizumab on Day 1 of 21-day cycles. In Cohort 1, 119 cisplatin-ineligible patients with locally advanced and metastatic UBC patients were enrolled to assess the efficacy of atezolizumab as a first-line treatment [86]. For a median follow-up of 17.2 months, the objective response rate was 23% (95% CI 16–31) and the complete response rate was 9%. In addition, the median PFS was 2.7 months and median OS was 15.9 months. In Cohort 2, 310 UBC patients who had disease progression during or following a prior platinum-based chemotherapy regimen were enrolled [87]. PD-L1 expression on tumor-infiltrating ICs was prospectively determined by immunohistochemistry, and all the patients were categorized into in three different groups based on percentage of PD-L1-positive ICs: IC0 (<1% expression), IC1 (≥1%, but ≤5% expression), and IC2/3 (≥5% expression). For patients with a minimum of 6 weeks of follow-up, the objective response rates were 26% (95% CI 18–36) in the IC2/3 group, 18% (95% CI 13–24) in the IC1/2/3 group, and 15% (95% CI 11–19) in all patients. In addition, the median OS were 11.4 months (95% CI 9.0 to not estimable) in the IC2/3 group, 8.8 months (95% CI 7.1–10.6) in the IC1/2/3, and 7.9 months (95% CI 6.6–9.3) in all patients. The results first demonstrated that atezolizumab was active in UBC. These results indicated that atezolizumab demonstrated encouraging durable response rates, survival, and tolerability, supporting its therapeutic use in untreated UBC.

In a Phase III trial IMvigor211 (NCT02302807), 931 patients with metastatic UBC who have previously failed platinum-based chemotherapy were enrolled and randomly assigned to either atezolizumab or chemotherapy (vinflunine, paclitaxel, or docetaxel) [88]. In the IC2/3 population (*n*=234), the OS and ORR were similar between the atezolizumab group and the chemotherapy group. However, the duration of response was remarkably longer in the atezolizumab group than in the chemotherapy group (median 15.9 months [95% CI 10.4 to not estimable] vs. 8.3 months [5.6–13.2]; HR 0.57, 95% CI 0.26–1.26). Safety analysis also favored atezolizumab with the lower high-grade toxicities (20 vs. 43%) and the lower incidence of treatment discontinuation (7 vs. 18%). In addition, 195 patients were classified into luminal (*n*=73) and basal (*n*=122) subtypes as according to the gene expression profile defined by TCGA. Surprisingly, the ORR in the luminal cluster II subtype (34%) was significantly higher than those in luminal cluster subtype I (10%), basal cluster subtype III (16%), and basal cluster subtype IV (20%). Furthermore, the median mutation load was significantly increased in responders (12.4 per Mb) compared with non-responders (6.4 per Mb). Taken together, atezolizumab was approved for patients with locally advanced or metastatic UBC who progressed on or after platinum-based chemotherapy (Figure 1).

3.2.1.4. Durvalumab

Durvalumab is another PD-L1 inhibitor that has been approved for treatment of advanced UBC that progressed during or after previous platinum-based chemotherapy. In a phase 1/2 multicenter and open-label study (NCT01693562), 61 patients with inoperable or metastatic UBC were recruited to assess the safety and efficacy of durvalumab [89]. All the patients (40 PD-L1-positive and 21 PD-L1-negative) were treated with durvalumab 10 mg/kg every 2 weeks for up to 12 months or until progression, or unacceptable toxic effects. The ORR was 31% in the overall population and 46.4% of the PD-L1 positive versus 0% in the PD-L1 negative subgroup. TRAEs were mostly mild, with no Grade 4 or 5 events occurring. Fatigue and diarrhea were most common (13.1% and 9.8%, respectively), and Grade 3 events occurred in only three (4.9%) patients.

In 2017, the updated results including 191 UBC patients were reported [90]. The ORR was 17.8% of patients, including complete response in seven patients. Specifically, the ORR in high PD-L1 expression group (28%) was significantly greater than that in low-or-negative PD-L1 expression group (5%). In addition, the median PFS and OS were 1.5 and 18.2 months, respectively, with the 1-year OS rate at 55%. High grade (Grades 3 or 4) TRAEs were noted in 6.8% of patients, and there were two treatment related deaths noted from autoimmune hepatitis and pneumonitis.

3.2.1.5. Avelumab

Avelumab is another anti-PD-L1 IgG1 monoclonal antibody. To assess the safety and antitumor activity of avelumab, 44 patients with refractory metastatic UBC were recruited in Multicenter, Phase I Study (NCT01772004) [91]. All the patients received avelumab 10 mg/kg intravenously every 2 weeks after platinum-based chemotherapy and unselected for PD-L1 expression. In the analysis process, PD-L1 positivity was defined as expression by immunohistochemistry on ≥5% of tumor cells. In a median of 16.5 months of follow-up, the ORR was 18.2% (95% CI, 8.2–32.7%; five complete responses and three partial responses), and seven of eight responding patients had PD-L1-positive tumors. In addition, the responses were ongoing in six patients (75.0%). The median PFS was 11.6 weeks (95% CI, 6.1–17.4 weeks), the median OS was 13.7 months (95% CI, 8.5 months to not estimable), and the 12-month OS rate of 54.3% (95% CI, 37.9–68.1%). Furthermore, the most frequent TRAEs of any grade were fatigue/asthenia (31.8%), infusion-related reaction (20.5%), and nausea (11.4%). Grades 3 to 4 TRAEs occurred in three patients (6.8%) including asthenia, AST elevation, creatine phosphokinase elevation, and decreased appetite.

In 2020, a Phase III clinical trial (NCT02603432) demonstrated that avelumab significantly improved survival in patients who developed the most common type of UBC. In that program, treatment with avelumab resulted in a 31% reduction in the risk of death and a median OS of 21.4 months, compared to 14.3 months for patients not treated with the drug [92]. Overall, avelumab showed strong antitumor activity with an acceptable safety profile and prolonged survival in patients with platinum-refractory metastatic UBC, with greater activity noted in PD-L1 positive tumors. These results accelerated the FDA approval for this indication.

#### 3.2.2. CTLA-4

Engagement of B7 ligands, notably B7-1 (CD80) and B7-2 (CD86), on antigen-presenting cells with CD28 co-stimulatory receptors on T cells stimulates the proliferation and activation of CTLs, which subsequently elicits an anti-tumor immune response in the host. This anti-tumor immunity is negatively regulated by CTLA-4 molecules. First discovered in 1987, CTLA-4 is a leukocyte differentiation antigen and a transmembrane receptor on T cells [29] (Figure 1). As CTLA-4 has a stronger binding affinity for B7 ligands than does CD28, CTLA-4 competitively inhibits the ligation of B7 and CD28, thereby attenuating T cell proliferation and activation, and suppressing anti-tumor immunity [45,46,72].

Genetic variations in the *CTLA-4* gene are negatively correlated with UBC [93,94]. Patients with UBC have a significantly lower frequency of the *CTLA-4* +49GG genotype and G allele compared with healthy controls [93]. Two CTLA-4 inhibitors, ipilimumab and tremelimumab, are currently used in immunotherapy for UBC [95]. In 2010, a Phase II trial of 12 patients with localized UBC demonstrated the benefit of ipilimumab as a neoadjuvant before radical cystectomy [96]. Further immunophenotyping and immunohistochemical analysis of UBC patient samples showed markedly increased frequencies of CD4^+^ and CD8^+^ T cells. However, Galsky *et al*. reported that ipilimumab did not significantly enhance response rate or 1-year OS in UBC patients compared with gemcitabine plus platinum-based chemotherapy [97]. Nonetheless, the response rate was significantly higher in patients with deleterious somatic DNA damage response mutations [97].

To enhance the ORR of immunotherapy, more and more combined treatment strategies have been developed. Combination immunotherapy with nivolumab and ipilimumab for locally advanced or metastatic UBC is under intensive investigation, which has proven to be effective in other forms of malignancy with potentiated cancer immune response with the dual-agent approach, followed by nivolumab maintenance therapy [98]. In an open-label phase II study CheckMate 032 (NCT01928394) [99], 274 patients with advanced or metastatic UBC previously treated with platinum-based chemotherapy were enrolled to investigated this regimen. Patients were randomly assigned to receive single-agent nivolumab 3 mg/kg (N group) or nivolumab 3 mg/kg plus ipilimumab 1 mg/kg (NI group) or nivolumab 1mg/kg plus ipilimumab 3 mg/kg, with the combinations followed by nivolumab 3 mg/kg maintenance therapy (NIN group). After a follow-up of 8 months, the ORR of NIN group, NI group, and N group was 38%, 27%, and 26%, respectively. The expression of PD-L1 did not influence the ORR. Furthermore, there was no statistically significant improvement in PFS or OS between groups [98].

In another phase III trial (DANUBE, NCT02516241), durvalumab was combined with tremelimumab as a first-line treatment for metastatic UBC patients [40,100]. 1032 patients were randomized in a 1:1:1 fashion to durvalumab monotherapy at 1.5 g IV every 4 weeks (D group) or durvalumab with tremelimumab at 75 mg IV every 4 weeks induction as 4 doses followed by maintenance durvalumab at 1.5 g IV every 4 weeks (DTD group) versus chemotherapy with gemcitabine and cisplatin or carboplatin for up to 6 cycles (C group), until disease progression or unacceptable toxicity. The median OS was not significantly different among D group, DTD group, and C group. The overall results of DANUBE were therefore considered negative for its primary endpoint.

**Figure 2:**
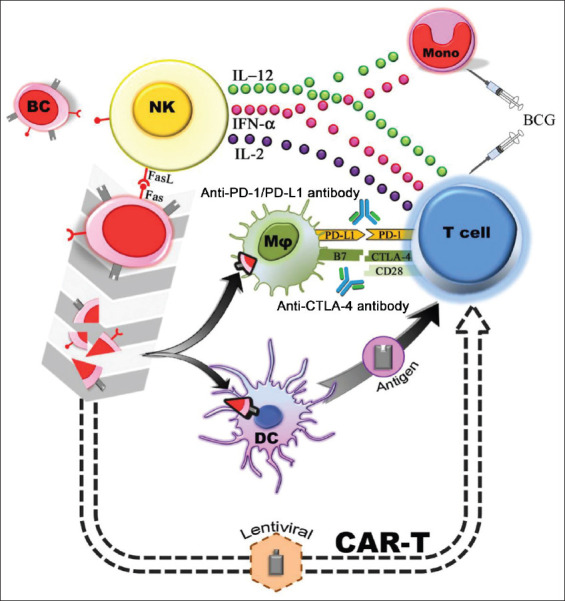
Immunotherapy strategies for urothelial bladder cancer

At present, five checkpoint inhibitors, atezolizumab, avelumab, durvalumab, nivolumab, and pembrolizumab, are available for the treatment of UBC [101] (Figure 2). However, some issues including side-effects and curative effect need to be addressed [102]. Therefore, further research and consideration of interventions such as combinational therapies are warranted to improve the clinical activities of PD-1/PD-L1 inhibitors in the future.

### 3.3. Cytokines

Cytokines are low-molecular-weight membrane-bound proteins that function as communicators between the host and ICs to regulate homeostasis of the immune system. Tumor cells secrete cytokines with tumor-promoting roles, such as uncontrolled cancer cell proliferation, anti-apoptosis features, metastasis progression, and initiation of immune escape of tumor cells [103]. Therapies such as interferon gamma (IFN-γ), IL-2, and tumor necrosis factor (TNF-α), have been approved for cancer treatment [103].

Various cytokines, including IFN-α [33], IFN-γ [34], IL-2 [37], and IL-12 [35], have been used in NMIBC therapy. In 1984, Pizza *et al*. [104] injected IL-2 directly into UBC tumors and found that 50% of the tumors disappeared (Figure 1). IL-12 therapy showed encouraging results in UBC treatment and its subcutaneous injection into an *in vivo* UBC model prolonged survival and reduced tumor growth [105]. Earlier, subcutaneous administration of IL-12 protected a mouse model from UBC relapse [36], and IL-12 therapy increased IFN-γ levels in serum and urine of mouse models. Besides its antiproliferative and pro-apoptotic activities, IFN-γ upregulated the expression of MHC-I, MHC-II, ICAM-I, B7-1, B7-2, and Fas on UBC cells, resulting in enhanced complement--mediated cytotoxicity [38] (Figure 2). However, despite these anti-tumor effects, cytokines did not show great advantage over BCG therapy.

### 3.4. Adoptive T cell immunotherapy

Adoptive T cell immunotherapy has the main goal of eliciting a T cell anti-tumor response in the tumor microenvironment. This therapy involves the *ex vivo* culture of T cells, which are reinfused back into cancer patients to initiate anti-tumor immunity.

In an open trial, Sherif *et al*. conducted adoptive T cell transfer in 12 patients with metastatic UBC [106]. First, the sentinel nodes were excised in conjunction with cystectomy, followed by extraction and *ex vivo* culture of T lymphocytes. Reinfusion of these T cells was successfully performed in six patients without any major adverse effects. These results indicated that adoptive immunotherapy based on T cells from tumor-draining lymph nodes was feasible in advanced UBC. Moreover, the Winqvist group further confirmed the feasibility of adoptive T cell transfer in metastatic UBC patients [107]. The diminished or obliterated nodal metastases were detected in two of nine patients, and the survival times of the two responders were 35 and 11 months, respectively. Taken together, these studies demonstrate the potential of adoptive T cell immunotherapy as an alternative therapeutic option.

In 2017, Shenzhen Geno-Immune Medical Institute carried out a Phase I/II and multicenter study including 20 locally advanced or metastatic UBC patients who have no further treatment available, to evaluate the efficacy and safety of 4SCAR-T cells (Figures 1 and 2, NCT03185468).

### 3.5. DCs

DCs are antigen-presenting cells that contribute to the initiation of T cell-mediated immune responses [108]. First discovered in the lymph nodes of mice by Steinman in 1973, DCs account for <1% of human PBMCs and are widely distributed throughout the body except in the brain [109]. DCs possess strong capabilities for antigen capture, processing, and presentation, and can activate resting T cells and promote T cell-dependent tumoricidal activities.

Tumor-specific antigen-sensitized DCs are also used in tumor immunotherapy. In general, tumor-specific antigens or antigenic peptides are used to sensitize DCs *in vitro* and they are then reinfused back into patients to stimulate the production of antigen-specific CTLs, resulting in a protective immune response and elimination of tumor cells [110]. Nishiyama *et al*. showed that a combination of melanoma antigens-3 (MAGE-3) antigen peptide (IMPKAGLLI) and HLA-A24-sensitized DCs possessed significantly elevated ability to induce a MAGE-3^+^ cell-specific CTLs response compared with MAGE-3-expressing UBC cells or non-pulsed DCs *in vitro* [111]. Four HLA-A24^+^ patients with advanced MAGE-3^+^ UBC were treated with injections of sensitized DCs every 2 weeks, a minimum of six and a maximum of 18 times. Three of the four patients showed significant reductions in size of lymph node metastases and/or liver metastases, with no significant untoward side-effects (Figure 2). However, UBC specific DCs based cancer immunity still needs to be further explored.

### 3.6. Macrophages

Macrophages are mononuclear white blood cells that originate from two sources: bone marrow-derived monocytes and tissue-resident macrophages. They are well-known for phagocytosis, in which macrophages engulf and digest cell debris and pathogens, and activate lymphocytes or other ICs [112,113]. In cancer, TAMs are closely related to prognosis in UBC [114-117].

As macrophages are crucial to UBC carcinogenesis, unraveling the mechanisms by which macrophages exert anti-tumor immunity could lead to novel therapeutic approaches. Thiel *et al*. found that the supernatant from BCG-stimulated macrophages elevated the expression of NOS2 and NO, which led to cell death in UBC cell line MBT2. A NOS-inhibitor, L-Nitro-arginine methyl ester, blocked NO-synthesis but did not affect cell death, suggesting that secondary stimulation from BCG-activated macrophages induced NO-independent cell death in UBC cells [63].

Liu and Duan found that *Pseudomonas aeruginosa*-mannose-sensitive hemagglutinin (PA-MSHA) promoted M1 polarization of TAMs via upregulating expression of M1-related genes such as IL-12, TNF-α, and IFN-γ, thereby enhancing their phagocytotic ability [118]. By contrast, PA-MSHA inhibited the M2 polarization of TAMs through downregulating expression of M2-related genes such as IL-4, IL-10, and TGF-β. PA-MSHA could also promote apoptosis and inhibit proliferation, invasion, and migration of mouse UBC cells by inducing M1 polarization. Furthermore, Tian *et al*. showed that a *Streptococcus*-derived anticancer immunotherapeutic agent, OK-432, inhibited proliferation, migration, and metastasis and induced apoptosis of T24 and EJ UBC cell lines *in vitro*. Mechanically, OK-432 inhibited the expression of IL-10 and promoted the expression of TNF-α in TAMs, resulting in enhanced anti-tumor capability [119]. Finally, Yang *et al*. established an orthotopic urinary UBC model by intravesical injection of MBT-2 cells [120]. They found that TAMs are closely related to lymph angiogenesis and lymphatic metastasis of UBC. Thus, macrophages serve as potential targets in the immune landscape of the UBC tumor microenvironment (Figure 2).

## 4. Discussion

Tumor immune escape is one of the ten features necessary for tumor development. The immune escape mechanisms of UBC were very complex, involving the participation of genes, metabolism, inflammation, vascularity and other aspects. Advances in molecular biology and high throughput sequencing have revealed the immune escape mechanisms of UBC, which commonly resulted from the transformation of tumor cells themselves or the alteration of tumor microenvironment. Based on those scientific progress, various novel immunotherapies have emerged as effective treatment for UBC after surgery, radiotherapy, chemotherapy, and targeted therapy. For example, various immune checkpoint blocking drugs such as pembrolizumab, nivolumab, atezolizumab, durvalumab and avelumab have been approved by the FDA and have achieved encouraging therapeutic results, expanding the traditional chemotherapy and surgery-based treatment for UBC.

The immunotherapy has a significant effect, improving the current status of UBC treatment. At present, the commonly used immunotherapy methods for UBC that have been clinically approved mainly include BCG and immune checkpoint blockades. BCG perfusion therapy is the standard post-operative perfusion regimen for intermediate-risk and high-risk NMIBC, reducing the recurrence rate of 32–56%. However, the mechanisms of BCG perfusion therapy are still under investigated. After BCG therapy, multiple tumors are the risk factors of UBC recurrence and high stage and grade are associated with tumor progression [121]. It has been shown that several biomarkers of UBC, such as p53, RB, survivin, B-cell lymphoma/leukemia 2, and fibroblast growth factor receptor, could be applied in the prediction the effect of BCG perfusion [121]. However, they have not been widely confirmed. Thus, the indicators to predict the effectiveness and novel therapy strategies to improve the effectiveness of BCG perfusion should be further studied.

Immune checkpoint blockades have improved the OS of patients with UBC. The approval of these drugs has improved the efficacy of advanced second-line UBC. The ORR increased from 12% to 27%, among which patients with positive PD-L1 expression had a higher ORR and the second-line median OS has increased from 7 months to 20 months [90]. In addition, the median OS of patients with first-line intolerance to cisplatin increased from <10 months to 19 months [86].

Immunotherapy has achieved desirable results in the treatment of UBC. The good tumor control effect and the lower incidence of TRAEs of immunotherapy have unique advantages compared with traditional chemotherapy. However, there are also some problems and challenges. Firstly, nearly 40% of NMIBC patients have failed BCG treatment [122], BCG treatment can cause inflammation, but the mechanism is not fully understood [123]. Secondly, checkpoint blockage still suffers from low response rates and high prices. Some studies have shown that only 12% of patients benefit from treatment with immune checkpoints [124], making it difficult to achieve satisfactory treatment outcomes. Finally, some clinical trials including single immune checkpoint inhibitors have failed to reach the research endpoints, such as atezolizumab’s IMvigor130 [125] and pembrolizumab’s KEYNOTE-361 [126], and the curative effect of single immune checkpoint was not better than chemotherapy, such as pembrolizumab’s KEYNOTE-361 [126].

Therefore, combination therapy could be investigated to improve treatment outcomes, such as the dual immune combination regimen of PD-1/PDL-1 inhibitors with CTLA-4 inhibitors. For example, Sharma *et al*. improved the ORR of patients with nivolumab plus ipilimumab combination therapy [99]. CTLA-4 inhibitors go to the source and increase the anti-cancer T-cell numbers, while PD-1 inhibitors act in peripheral blood or tumors, allowing the binding process of PD-1 to PD-L1 to be blocked in these viable agents, thus freeing ICs to kill and launch a strike against the tumor [127]. Another combination strategy is composed of immune checkpoint blockades and chemotherapy. For example, the combination of pembrolizumab, and gemcitabine and cisplatin or carboplatin enhanced the ORR, median PFS and median OS of UBC patients compared to those of chemotherapy or pembrolizumab [82].

Cisplatin-based chemotherapy has been the standard of care for UBC for the past 30 years. The emergence of anti-PD-1/PD-L1 treatment in UBC clinical settings suggests that immunotherapy is a promising therapeutic approach for this disease. However, the complex interplay between immune escape of UBC cells and the immune system remained largely unknown. Greater understanding of the mechanisms of UBC development, the molecular characteristics of UBC, and its immune landscape will improve the efficacy of immunotherapy, enabling novel and robust immunotherapies to be developed with the goal of eradicating UBC.
